# One respiratory cycle as a minimum time unit for making behavioral decisions in the mammalian olfactory system

**DOI:** 10.3389/fnins.2024.1423694

**Published:** 2024-09-09

**Authors:** Kensaku Mori, Hitoshi Sakano

**Affiliations:** ^1^RIKEN Center for Brain Science, Wako, Saitama, Japan; ^2^Department of Brain Function, School of Medical Sciences, University of Fukui, Matsuoka, Japan

**Keywords:** sensory inputs, olfactory system, behavioral decisions, voluntary behaviors, inhalation and exhalation, one respiratory cycle-one decision making

## Abstract

Voluntary behaviors such as sniffing, moving, and eating require decision-making accompanied by intentional respiration. Based on the study of respiration-coherent activity of rodent olfactory networks, we infer that during the inhalation phase of respiration, olfactory cortical areas process environmental odor information and transmit it to the higher multisensory cognitive areas via feedforward pathways to comprehensively evaluate the surrounding situation. We also infer that during the exhalation phase, the higher multisensory areas generate cognitive-signals and transmit them not only to the behavioral output system but also back to the olfactory cortical areas. We presume that the cortical mechanism couples the intentional respiration with the voluntary behaviors. Thus, in one respiratory cycle, the mammalian brain may transmit and process sensory information to cognize and evaluate the multisensory image of the external world, leading to one behavioral decision and one emotional expression. In this perspective article, we propose that one respiratory cycle provides a minimum time unit for decision making during wakefulness.

## Introduction

During wakefulness, we detect environmental information via the visual, olfactory, auditory, gustatory, and somatosensory systems. When the surrounding situation is comfortable and satisfactory, we become happy and relaxed trying to maintain the present state. In contrast, when the sensory systems detect negative changes in the environment, we become stressed, and take action to avoid the danger or to remove it for our survival. After detecting the environmental signals, the higher-order multisensory cognitive-areas, such as the prefrontal cortex and amygdala, predict the upcoming situation, whether it is going to be good (positive) or bad (negative) for us.

**Figure 1:**
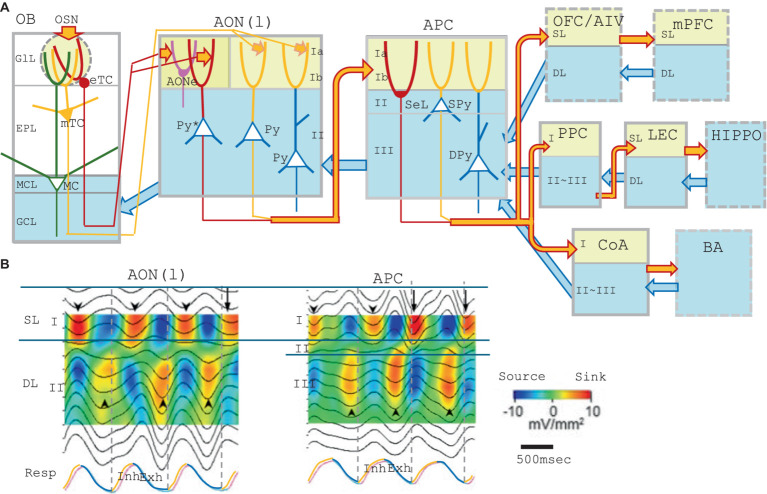
**(A)** A schematic diagram of the central olfactory areas. The presumed feedforward transmission of external odor information (during inhalation) and possible top–down transmission of cognitive signals (during exhalation) are shown. Orange arrows show the feedforward transmission of external odor signals from the lower cortical area to the superficial layer (yellow) of the higher cortical area during inhalation. Blue arrows are for the top–down transmission of cognitive signals from the higher cortical area to the deep layers (blue) of the lower cortical area during exhalation. Olfactory cortical areas (solid gray boxes) include the olfactory bulb (OB), anterior olfactory nucleus pars externa (AONe), anterior olfactory nucleus pars lateralis [AON(l)], anterior piriform cortex (APC), posterior piriform cortex (PPC), lateral entorhinal cortex (LEC), and cortical nucleus of amygdala (CoA). Higher-order multisensory cognitive areas (broken line boxes) include the orbitofrontal cortex (OFC), ventral agranular insular cortex (AIV), medial prefrontal cortex (mPFC), and basal amygdaloid nucleus (BA). Abbreviations for the layers: GlL, glomerular layer; EPL, external plexiform layer; MCL, mitral cell layer; GCL, granule cell layer; Ia, layer Ia; Ib, layer Ib; II, layer II; III, layer III; SL, superficial layer; and DL; deep layer. Abbreviations for the cells: eTC, external tufted cell; mTC, middle tufted cell; MC, mitral cell; Py, pyramidal cell; Py*, pyramidal cell of the AON(l) that projects apical dendrites to the layer Ia of the AONe; SeL, semilunar cell; SPy, superficial pyramidal cell; and DPy, deep pyramidal cell. **(B)** Depth profiles and current-source-density analysis of local field potentials recorded at intervals of 100 μm in depth in the AON(l) and APC of the awake resting rats. The raw local field potential was band-pass filtered (0.5–3 Hz) and current-source-density was calculated. Warm colors indicate the current-sink, while cold colors show the current-source. Respiration was monitored by a thermocouple placed at the nostril (RESP, shown in the bottom trace). The inhalation phase (Inh) is in orange, while exhalation phase (Exh) is in blue. Only slow-wave current-sinks are shown, but not the β-and γ-range fast oscillatory current-sinks, because of the band-pass filtering. Downward arrowheads indicate slow-wave current-sinks in the superficial layer while upward arrowheads indicate slow-wave current-sinks in the deep layer. The figures are modified from Narikiyo et al. (2018).

In the mouse olfactory system, basic information for survival, e.g., in searching food, detecting danger, and finding mating partners, is processed by hard-wired neural circuits. For these innate decisions, odor signals are directly transmitted from the olfactory bulb (OB) to the valence regions in the amygdala via hard-wired neural circuits (direct pathways) (Miyamichi et al., 2011; Root et al., 2014; Inokuchi et al., 2017). Such instinctive decisions are stereotyped and genetically determined as a result of natural selection during evolution. In addition, sensory decisions are also made based on the olfactory memory of previous scene by learning. Odor information represented as an odor map, a pattern of activated glomeruli in the OB, is transmitted mainly by tufted cells to the anterior olfactory nucleus (AON), preserving the map topography (Mori and Sakano, 2021). Especially, external tufted cells (eTCs) in the OB project axons keeping their topographic orders to the layer Ia of the AON pars externa (AONe) (Yan et al., 2008; Hirata et al., 2019). It has been reported that at least part of the AON pars lateralis [AON(l)] receives topographically organized inputs, since apical dendrites of pyramidal cells of the underlying AON(l) (Figure 1A, marked by Py*) penetrate into the layer Ia of the AONe (Scott et al., 1985). Individual neurons in the AON(l) selectively respond to specific odorant categories (Kikuta et al., 2008) that are topographically organized in the OB.

The odor map appears to be utilized as a QR code (two-dimensional bar code) in searching for the memory engram of the associated scene in the previous odor experience (Wilson and Sullivan, 2011; Russo et al., 2020; Chen et al., 2022; Poo et al., 2022). For decision-making, the recollected memory scene further activates the valence circuit once connected to a particular valence region in the olfactory amygdala (multi-synaptic pathways) (Motanis et al., 2014; Janak and Tye, 2015; Jin et al., 2015; East et al., 2021).

It is notable that environmental odor information is detected only during the inhalation phase of respiration. The orthonasal odor signals seem to be processed during inhalation to make a decision of how to respond to the surrounding situation. Olfactory responses are triggered in the following exhalation phase directed by the sensory decision/cognition that is made during the inhalation-exhalation transition phase. Emotional expression and behavioral responses are generally induced during the exhalation phase, as seen in vocal communication. In the language conversation in humans, talking occurs in synchrony with a slow and long exhalation, and the duration of exhalation varies as a function of the utterance produced over the course of the exhalation phase. A speaker takes a short breath (inhalation) at the end of a simple sentence (Rochet-Capellan and Fucks, 2013).

External sensory information receives more attention during the inhalation phase. In contrast, behavioral and emotional responses are induced in the exhalation phase not only in the olfactory system but also in other sensory systems. This is probably because decision making takes place by integrating all valence decisions of other sensory systems together with the olfactory decision that is correlated with the respiratory cycle. Based on the studies of respiration-coherent activity of neural networks, we propose that the inhalation phase provides a timeframe for the feedforward transmission of environmental odor signals through the OB and the olfactory cortex (OC) to the higher-order multisensory cognitive-areas. We also assume that during exhalation, the higher-order cognitive-areas generate cognitive signals for decision making, and transmit the signals not only to the motor output system but also back to the OC and OB. Thus, one respiration cycle appears to provide a minimal time unit for decision making. In this perspective article, we discuss the recent progress in the study of sensory and behavioral decision-making in relation to the inhalation and exhalation phases of respiration.

## Feedforward transmission of odor information during inhalation

When an animal is awake, the cerebral cortex constantly receives visual, auditory, and somatosensory information from the environment. In the olfactory system, external/orthonasal odor information is received only during the inhalation phase of the respiratory cycle, thus the cerebral cortex is isolated from the environmental odor information during the exhalation phase. It appears that the inhalation phase provides a timeframe for the central olfactory areas, e.g., the OB, lateral part of AON [AON(l)], anterior piriform cortex (APC), posterior piriform cortex (PPC), cortical amygdala (CoA), and lateral entorhinal cortex (LEC) (Figure 1A), to process odor information from the surrounding world (Mori and Sakano, 2021, 2022, 2024). The respiration-phase may play a key role in coordinating the timing of feedforward transmission of external odor information across multiple areas in the central olfactory cascades; OB → AON(l) → APC → PPC → LEC, or OB → AON(l) → APC → CoA (Figure 1A). As the central olfactory cascades project to the higher-order multisensory cognitive areas such as the orbitofrontal cortex (OFC), ventral agranular insular cortex (AIV), medial prefrontal cortex (mPFC), hippocampus (HIPPO), and basal nucleus of amygdala (BA) (Price, 1985; Ekstrand et al., 2001; Chen et al., 2014), we further speculate that the respiration-phase also determines the timing of feedforward transmission of external odor information to the higher-order multisensory cognitive areas beyond the central olfactory cascades.

We previously reported that external tufted cells (eTCs) and middle tufted cells (mTCs) in the lateral OB map appear to show synchronized burst firing and transmit olfactory sensory signal to layer Ia of the AONe and AON(l) during the inhalation phase (Figure 1A) (Mori and Sakano, 2021, 2022, 2024). The pyramidal cells in the AON(l) that receive the olfactory signal in layer Ia project their axons to layer Ib of APC and depolarize the apical dendrites of APC pyramidal-cells (Haberly and Price, 1978b; Luskin and Price, 1983; Russo et al., 2020).

Current-source-density analysis of local field potentials in the AON(l) in the awake resting rats demonstrate β-and γ-range fast oscillatory current-sinks and a slow-wave current-sink in the superficial layer (SL, layer I) during the inhalation phase (Figure 1B, downward arrowheads) (Narikiyo et al., 2018). Sensory deprivation experiments suggest that the inhalation-phased olfactory input from eTCs and mTCs drives the fast oscillatory current-sinks in the SL and induces inhalation-phased burst firings of pyramidal cells in the AON(l) (Narikiyo et al., 2018). In contrast, the slow-wave current-sink in the SL occurs during the inhalation phase even in the absence of the olfactory input (Narikiyo et al., 2018). These results suggest that the brain internally and spontaneously generates the slow-wave depolarization in the SL dendrites of AON(l) pyramidal-cells during the inhalation phase.

The AON(l) pyramidal-cells project their axons to layer Ib of the APC. Current-source-density analysis of local field potentials in the APC demonstrates β-and γ-range fast oscillatory current-sinks and a slow-wave current-sink in the SL (layer I) during the inhalation phase in the awake rats (Figure 1B, APC). Sensory deprivation experiments suggest that the inhalation-phased olfactory input drives the fast oscillatory current-sinks in the SL and induces burst firings of APC pyramidal-cells during the inhalation phase. On the other hand, the slow-wave current-sink in the SL occurs during the inhalation phase even in the absence of the olfactory input (Narikiyo et al., 2018), suggesting that the brain internally and spontaneously generates the slow-wave depolarization in the SL dendrites of APC pyramidal-cells during the inhalation phase.

The APC pyramidal-cells project their axons to layer Ib of the PPC (Haberly and Price, 1978a). Current-source-density analysis in the PPC also indicates that the olfactory sensory input drives the fast oscillatory current-sinks in the SL during the inhalation phase. However, a slow-wave current-sink in the SL occurs even in the absence of the olfactory input, suggesting that the slow-wave current-sink in the SL is generated internally and spontaneously by the brain during the inhalation phase.

These results may suggest the possibility that the slow-wave depolarization in the SL dendrites is generated internally and intentionally in synchrony with the inhalation phase in the AON(l), APC, and PPC, independently from the inhalation-phased olfactory sensory input. This idea is consistent with the observation that the slow-wave current-sink sometimes starts before the onset of the inhalation phase (Figure 1B, downward arrows).

It is not clear yet which neural circuit drives the slow-wave depolarization in the SL dendrites of pyramidal cells in synchrony with the inhalation phase of respiration. One possibility is that a subset of GABAergic cells in the basal forebrain (BF) generates burst discharges during the intended inhalation phase and send inhibitory axons to the layer-I inhibitory interneurons such as neurogliaform cells and horizontal cells (Suzuki and Bekkers, 2012; Suzuki et al., 2022) in the AON(l), APC, and PPC. The BF GABAergic input may inhibit the layer-I inhibitory interneurons, thus disinhibit the SL dendrites of pyramidal cells, resulting in the slow-wave current-sink in the SL during the intended inhalation phase. We speculate that the slow-wave depolarization in the SL dendrites during the inhalation phase may boost the transmission of olfactory sensory signals to the pyramidal cells in the AON(l), APC, and PPC, resulting in the effective feedforward transmission of olfactory signals through the successive stages of the central OC cascades (Figure 1A, orange arrows) (Mori et al., 2013). We thus propose that one inhalation provides a minimal time unit for the feedforward transmission of external odor information.

## Top–down transmission of cognitive information during exhalation

Respiration-phase-coherent activity occurs not only in the SL but also in the deep layer (DL) of the AON(l), APC, and PPC (Figure 1B). Current-source-density analysis of local field potentials in these areas demonstrates fast oscillatory current-sinks and a slow-wave current-sink in the deep layer (DL, layer II in the AON, and layers II/III in the APC and PPC) during the exhalation phase (Figure 1B, up-facing arrowheads). Sensory deprivation experiments demonstrate that even in the absence of the olfactory sensory input, the fast oscillatory current-sinks occur in the DL, and that pyramidal cells show increased firings during the exhalation phase (Narikiyo et al., 2018). Sensory deprivation experiments also demonstrate that the slow-wave current-sink in the DL occurs during the exhalation phase even in the absence of the olfactory input. These results indicate that the brain internally and spontaneously drives a slow-wave depolarization in the DL dendrites of pyramidal cells during the exhalation phase.

It is yet to be studied which neural circuit generates the slow-wave depolarization in the DL dendrites. One possibility is that a specific subset of BF GABAergic neurons may show burst firings during the intended exhalation phase and send their axons to the inhibitory interneurons in the DL of the AON(l), APC, and PPC (Suzuki and Bekkers, 2012). The burst firings of the BF GABAergic neurons may inhibit the inhibitory interneurons, thus disinhibit the DL dendrites of pyramidal cells during the intended exhalation phase.

In the absence of the olfactory sensory input, pyramidal cells in the AON(l) and APC show diminished firing during the inhalation phase but increased firing during the exhalation phase (Narikiyo et al., 2018). In the case of APC, top–down inputs from the amygdala, OFC, AIV, and PPC mainly terminate in the DL (Figure 1A, blue arrows) (Haberly and Price, 1978a; Illig, 2005), suggesting that the fast oscillatory current-sinks in the DL during exhalation partly reflect the activity of the top–down inputs from these higher cortical areas. Although it is unclear which neural circuit generates the exhalation-phased slow-wave depolarization in the DL dendrites, the slow-wave depolarization may facilitate the top–down signals to activate the APC pyramidal cells.

We thus hypothesize that the AON(l), APC, and PPC coordinately perform the respiration-phase-coherent switch of biasing between the feedforward olfactory-sensory signals and top–down signals. The inhalation phase is a time window for boosting the external odor inputs that arrive in the SL-dendrites of pyramidal cells, whereas the exhalation phase is for boosting the top–down signals to the DL dendrites, which originate from the higher cortical areas. It is possible that during exhalation, the amygdala feedbacks a valence signal via the top–down pathway to the DL of the APC, and that the mPFC and OFC feedback a behavioral decision-signal via their top–down pathways to the DL of APC. We presume that during exhalation, the higher multisensory areas transmit the cognitive information not only to motor output systems but also back to the olfactory cortex areas possibly to encode odor-behavior association memory.

Current-source-density analysis of the local field potential in the OB indicates that environmental odor inputs from olfactory sensory neurons occur in the superficial glomerular layer during the inhalation phase, whereas top–down signals from the AON and APC mainly arrive at the deep layer during the exhalation phase (Mori and Sakano, 2024). Therefore, the OB, AON(l), APC, and PPC may coordinately transmit and process the environmental odor information during the inhalation phase via the feedforward afferent pathways that terminate in the superficial layer of each area (Figure 1A, orange arrows). As the APC pyramidal-cells project to the OFC/AIV and the OFC pyramidal-cells project to the mPFC, we speculate that the environmental odor signals that arrive in the APC are further transmitted to the OFC/AIV and mPFC during the inhalation phase. Thus, in all the areas of the feedforward cascade of OB → AON(l) → APC → OFC/AIV → mPFC (Figure 1A, orange arrows), the brain may coordinate the timing of transmission and processing of environmental odor information with the inhalation phase. On the contrary, in all the areas of the top–down cascade of mPFC → OFC/AIV → APC → AON(l) → OB, the brain may coordinate the timing of transmission and processing of top–down cognitive signals with the exhalation phase (Figure 1A, blue arrows).

By the same token, in all the areas of OB ↔ AON(l) ↔ APC ↔ PPC ↔ LEC ↔ Hippo cascades, the brain may coordinate the timing of transmission and processing of environmental odor information with the inhalation phase, and top–down cognitive information with the exhalation phase. In all the areas of the OB ↔ AON(l) ↔ APC ↔ CoA ↔ BA cascades, the brain appears to coordinate the timing of transmission and processing of environmental odor information with the inhalation phase, and top–down valence information with the exhalation phase.

## One respiratory cycle and vocal communication

**Figure 2:**
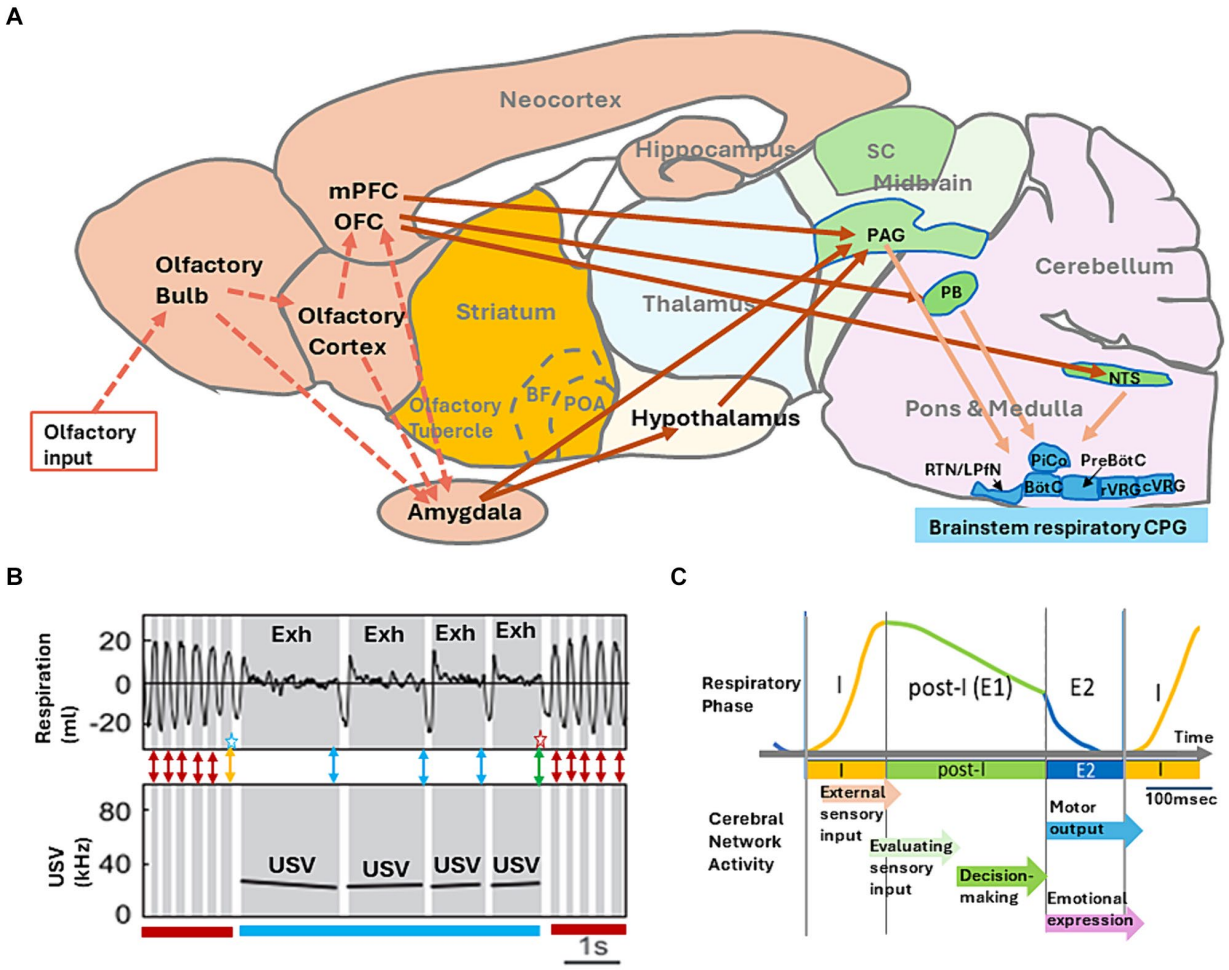
**(A)** A model of neural networks that may mediate behavioral decision-making. Information flow for external odors (broken arrows) and neural networks that couple the intentional respiration with the voluntary behaviors (solid arrows) are shown. Abbreviations: BF, basal forebrain; BötC, Bötzinger complex; cVRG, caudal ventral respiratory group; LPfN, lateral parafacial nucleus; NTS, nucleus of the solitary tract; OFC, orbitofrontal cortex; PAG, periaqueductal gray; PB, parabrachial-Kölliker-Fuse complex; POA, preoptic area; PreBötC, pre-Bötzinger complex; RTN, retro-trapezoid nucleus; rVRG, rostral ventral respiratory group; and SC, superior colliculus. **(B)** Simultaneous recording of respiratory signals (top panel, monitored by a plethysmography) and ultrasonic-vocalization (USV) signal (bottom panel, shown in a spectrogram) in an adult rat during the freezing behavior. The inspiratory phase is shown in white, and expiratory phase (Exh) in gray. The figure is modified from Boulanger-Bertolus et al. (2017). Double-headed red arrows indicate a possible timing of decision making to maintain the current behavioral strategy of freezing without USV. Double-headed blue arrows indicate a possible timing of decision making to maintain the current behavioral strategy of freezing with USV. A double-headed orange arrow with a blue star indicates a possible timing of decision making to change the behavioral strategy from freezing without USV to freezing with USV. A double-headed green arrow with a red star indicates a possible timing of decision making to change the behavioral strategy from freezing with USV to freezing without USV. **(C)** A schematic diagram illustrating the “one respiratory cycle – one behavioral decision making” hypothesis. I, inspiratory phase; post-I, post-inspiratory phase; E1, stage 1 expiratory phase; and E2, stage 2 expiration phase. Each arrow indicates a possible timing of cortical network function in relation to one respiratory cycle.

Vocal communication to conspecific animals is a voluntary social behavior and vocal emission is coordinated with exhalation (Sweeney and Kelley, 2014; Zhang and Ghazanfar, 2020; Demartsev et al., 2022). For this coordination, brainstem neural-circuits that control vocalization are coupled with the respiratory central pattern generator (CPG) (Barkan and Zornik, 2020). Figure 2B shows the ultrasonic vocalization (USV) of rats during freezing behavior induced by mild foot-shock (Boulanger-Bertolus et al., 2017). The USV of 22 kHz emitted by the freezing rat induces immobility responses also in the surrounding rats, suggesting that the foot-shocked rat communicates its fear via USV (Atsak et al., 2011; Boulanger-Bertolus and Mouly, 2021).

During shock-induced freezing, rats show either a train of regular ~4 Hz respirations without USV (Figure 2B, freezing without USV indicated by a dark red bar) or a train of very slow respirations with the 22 kHz USV (Figure 2B, freezing with USV indicated by blue bar). It should be noted that during freezing with USV, rats generate the vocal emission strictly in phase with exhalation, although the USV pauses in the phase of inhalation. This may suggest that one respiratory cycle provides a unit timeframe for the brain to prepare and generate one vocal communication. Namely, the exhalation phase is to generate one vocal emission and the subsequent inhalation phase is to prepare the next USV.

Tight coupling of one vocal emission and one exhalation phase indicates that the rat brain needs to decide in the late exhalation phase whether to maintain the current USV behavior for the next respiration cycle (Figure 2B, a blue double-headed arrows) or to change the behavioral strategy and terminate USV (Figure 2B, green double headed arrow with a red star). We speculate that during freezing without USV, the rat brain may need to decide in the late exhalation phase whether to maintain the current behavioral strategy of no USV (Figure 2B, red double-headed arrows) or to change the strategy to generate USV (Figure 2B, an orange double-headed arrow with a blue star).

Tight coupling of vocal communication and exhalation phase is widely observed in mammals including humans (Sweeney and Kelley, 2014; Zhang and Ghazanfar, 2020; Demartsev et al., 2022). We hypothesize that one respiratory cycle provides a minimum time unit for the mammalian brain to make vocal communication. Further studies will shed light on the basic neural mechanisms of language communication in relation to the respiratory cycle.

## One respiratory cycle and one behavioral decision-making

How does the behavioral decision-making correlate with the respiratory cycle? Neural circuits in the respiratory CPG of the brainstem (Figure 2A) organize the respiratory cycle that consists of inspiratory phase (I), post-inspiratory phase (post-I), and subsequent stage 2 expiratory phase (E2) (Figure 2C) (Smith et al., 2009; Krohn et al., 2023). The pre-Bötzinger complex (PreBötC) of respiratory CPG contains neural circuits for driving the phase I, activating the downstream inspiratory motoneurons to inhale the external air into the lung. As the inspiration also causes the inhalation of external odorants into the nasal cavity, detection of external odorants by olfactory sensory neurons occurs during phase I.

The post-inspiratory complex (PiCo) of respiratory CPG generates the phase post-I, terminating the activity of inspiratory motoneurons, and thus causes passive expiration. The phase post-I is also referred to as stage 1 expiratory phase (E1). At the end of the phase post-I, the lateral parafacial nucleus (LPfN) of respiratory CPG triggers the active exhalation phase (E2) by activating expiratory motoneurons. As the expiration during the phases E1 and E2 causes transportation of odorants via the retronasal olfactory route of mouth, pharynx, and lung into the nasal cavity (Shepherd, 2017), detection of retronasal/internal odorants occurs during the exhalation phase.

Voluntary behaviors such as moving, sniffing, eating, and sitting accompany intentional respiration. Because of the top–down multi-synaptic pathways starting from the mPFC, OFC, and amygdala to the brainstem respiratory CPG via the midbrain PAG (periaqueductal gray) (Figure 2A), prefrontal and amygdala networks may instruct the respiratory pattern necessary to perform the intended behavior (Foyd et al., 2000; Subramanian and Holstege, 2014; Warden et al., 2012; Franklin et al., 2017; Dampney, 2018; Hiser and Koenigs, 2018; Johnson et al., 2022). For example, rodents demonstrate a train of fast respirations (sniffing of 6–12 Hz) with short inhalation and exhalation phases in the translation movements. During the exploratory behavior in situations that require attention to the external world, rodents show fast respirations (sniffing of 3–8 Hz) with the high nasal flow rate coordinated with orofacial movements for the rapid sampling of external sensory information (Oka et al., 2009; Cury and Uchida, 2010; Kleinfeld et al., 2014; Kurnikova et al., 2017). In contrast, rodents demonstrate slow respiratory cycle with the long post-I phase during the resting and quiet sitting behavior.

As discussed above, during the foot-shock induced freezing-behavior, rodents are immobile showing the regular ~4 Hz respiration constituting of the small short I phase and the long exhalation phase. In the freezing with an alarm call of 22-kHz, USV occurs in phase with very long exhalation (Hegoburu et al., 2011; Moberly et al., 2018). During eating, rodents demonstrate slow respiration with a very long exhalation phase and occasional apnea at the timing of swallowing. Thus, the prefrontal and amygdalar control of respiration phases appears to play a key role in the successful execution of the intended behavior.

Respiration phase-coherent neural activities occur not only in the olfactory system but also throughout the brain including the mPFC (the anterior cingulate cortex, prelimbic cortex, and infralimbic cortex), OFC, and amygdala (Ito et al., 2014; Chi et al., 2016; Biskamp et al., 2017; Zhong et al., 2017; Herrero et al., 2018; Köszeghy et al., 2018; Moberly et al., 2018; Tort et al., 2018; Bagur et al., 2021; Girin et al., 2021; Kluger and Gross, 2021; Karalis and Sirota, 2022; Folschweiller and Sauer, 2023). Neural circuits in the mPFC, OFC, and amygdala may receive the information of current respiration phase from respiration phase-coherent activity of the central olfactory cortical areas and the corollary discharges of respiratory CPG. The mPFC and OFC are essential for making behavioral decisions (Kepecs et al., 2008; Warden et al., 2012, McLaughlin et al., 2021; Mair et al., 2022; Diehl and Redish, 2023) and mPFC axons project to the midbrain PAG and brainstem nuclei (parabrachial nucleus and nucleus of the solitary tract) connecting to the respiratory CPG (Foyd et al., 2000; Dampney, 2018). Thus, we propose that mPFC decision-making networks send the top–down signals to respiratory CPG, instructing the timing, magnitude, and duration of upcoming inhalation/exhalation to coordinate with the generation of intended future behavior (Figure 2A).

Even a simple decision-making operation requires the orchestration of numerous cognitive processes, multiple evaluation processes, and an action-choice process (Schall, 2005; Gallivan et al., 2018; Juavinett et al., 2018). Based on the above discussion, we would like to propose a hypothetical rule of “one respiratory cycle – one decision making.” This rule predicts that one respiratory cycle provides a minimum time unit for the brain to make one behavioral decision during wakefulness (Figure 2C). In this rule, the brain attends to the external events and actively samples external sensory information during the inhalation phase (phase I), generating the multi-sensory image of the current external world. During the subsequent post-I phase (passive exhalation phase), the brain evaluates the image of the current external world, changes its attention to the internal world, and then processes the sensory information of the self-body. We speculate that in the late exhalation phase, the brain decides whether to maintain the current behavioral strategy or change it to a new strategy to cope with the new environmental situation. During the active exhalation phase (E2), the brain executes the intended motor plan and expresses emotions.

Thus, one respiratory cycle provides a basic time unit to generate the integrated image of the behavioral scene that leads to decision-making and then to execute the behavioral output. The “one respiratory cycle – one decision making” hypothesis will give new insight into our understanding of neural circuit dynamics in the multi-sensory cognitive processes and behavioral decision-making.

## Discussion

When we are awake, our sensory systems are constantly detecting environmental changes in the outside world. We also detect the internal information from our body, including the physiological condition such as hunger and sickness. We evaluate whether our current situation is reasonable and satisfactory to us. When the brain is relaxed, various thoughts come to mind by spontaneous firing of memory engrams (Behrendt, 2013). We ask ourselves whether we are satisfied or not. When we feel that the situation is on the negative side from what it should be, we take action to improve it. In any case, we evaluate the surrounding situation and make a decision for behavioral and emotional responses. Therefore, decision making requires the temporal coordination of sensory-motor network-activity across the widely distributed areas in the brain, e.g., the cerebral cortex including exteroceptive and interoceptive sensory-cortex areas, multisensory cognitive-areas such as the amygdala and prefrontal cortex, and motor output areas. It has been proposed that the respiratory cycle acts as an oscillatory pacemaker, persistently coupling with the distributed brain-circuit dynamics (Karalis and Sirota, 2022; Tort et al., 2018; Folschweiller and Sauer, 2023).

In this perspective article, we have discussed a hypothesis that “one respiratory cycle is a minimum time unit for behavioral decision” in the mammalian olfactory system. We infer that during the inhalation phase, the brain transmits the external odor information to the higher multisensory cognitive-areas through the central olfactory cascades. Rodents increase the sniff rate up to 6–12 Hz during the translation movements and active explorative behaviors. During the fast continuous sniffs, however, the behavioral decision does not seem to occur in each sniff. The fast sniffs typically consist of several cycles of ‘inhalation-short pause’ followed by a long exhalation. We speculate that whole cycles of ‘inhalation-short pause’ consist of one inhalation phase and that decision making takes place during the subsequent long exhalation.

During the exhalation phase, the higher multisensory-areas appear to transmit the cognitive information not only to the motor output-system but also back to the central olfactory areas. In the exploratory behavior, respiration-phase-coherent operation of neural networks occurs not only in the olfactory system but also in the barrel field of the somatosensory cortex, auditory cortex, and visual cortex (Ito et al., 2014; Tort et al., 2018; Folschweiller and Sauer, 2023). This may suggest that respiration-phase-coherent operation takes place in various areas of the brain for processing the sensory information coming from the external world (Perl et al., 2019).

The respiratory CPG (central pattern generator) in the brainstem autonomously controls respiration. However, respiration is also under cortical volitional control, as evident from the voluntary respiration during human vocal communication and emotional expression (McKay et al., 1985; Herrero et al., 2018; Kluger and Gross, 2021). Initiation of intended motor outputs typically occurs in synchrony with the exhalation phase. For example, vocal emission requires the exhalation-phase-coherent activity of the cortex. We extend our inference about the central olfactory networks to the entire cortical networks including the prefrontal networks, which are essential for adjusting the animal’s behavior to its environment (Franklin et al., 2017; Johnson et al., 2022). Thus, we would like to propose that one respiratory cycle provides a minimal time unit of one behavioral scene, leading to one behavioral decision-making for the upcoming respiratory cycle.

As the cerebral-network activity synchronizes with the respiratory cycle during wakefulness, one respiratory cycle appears to provide a time frame for the network to interact with the external world. If a new visual event of behavioral significance occurs in the middle of exhalation, the cortical network may actively reset the respiratory cycle and try to cognize the visual event for behavioral decision making.

In contrast, during slow-wave sleep, the network activity occurs internally and independently of the respiratory cycle. Therefore, it is possible that the cerebral network does not interact with the external world during the slow-wave sleep. We propose that cortical control of the respiratory-phase progression is tightly linked to the cortical mechanisms for the sequential progression of multisensory cognition of the external world, behavioral decision making, and motor/emotional outputs. We also propose that the cortical control of respiratory-phases is coupled with the progression of feedforward processing of external information and the top–down encoding of the association memory of sensory input—behavioral decision.

In behaving animals, respiratory phases are currently monitored by a surgically implanted thermistor in the nasal cavity or by a plethysmography chamber. If more convenient methods are developed for monitoring the respiration, it will become possible to elucidate the relationship between respiration phases and large-scale cortical network activities in awake behaving animals. In this perspective article, we have discussed decision making in relation to the respiratory cycle mainly based on the recent studies on the rodent olfactory system. It will be interesting to analyze whether similar informational processing can be found in other sensory systems. Respiratory-phase related activation may be found in various brain activities; emotional expression, strategy thinking, recollecting memory, social communication, fear responses, indecision/hesitation, observation, and so on. Future studies of cortical-network dynamics in relation to the respiratory-phase progression will provide us with critical clues for understanding how our perceptions and decision makings arise from the cortical networks.

## Data Availability

The original contributions presented in the study are included in the article/supplementary material, further inquiries can be directed to the corresponding authors.

## Glossary

AIV: Ventral agranular insular cortex
AON: Anterior olfactory nucleus
AONe: Anterior olfactory nucleus pars externa
AON(l): Anterior olfactory nucleus pars lateralis
APC: Anterior piriform cortex
BA: Basal nucleus of amygdala
BF: Basal forebrain
CoA: Cortical amygdala
CPG: Central pattern generator
DL: Deep layer
eTC: External tufted cell
E1: Stage 1 expiratory phase of respiration
E2: Stage 2 expiratory phase of respiration
HIPPO: Hippocampus
I: Inspiratory phase of respiration
LEC: Lateral entorhinal cortex
LPfN: Lateral parafacial nucleus
mPFC: Medial prefrontal cortex
mTC: Middle tufted cell
NTS: Nucleus of the solitary tract
OB: Olfactory bulb
OC: Olfactory cortex
OFC: Orbitofrontal cortex
PAG: Periaqueductal gray
PBN: Parabrachial nucleus
PiCo: Post-inspiratory complex
Post-I: Post-inspiratory phase of respiration
PPC: Posterior piriform cortex
PreBötC: Pre-Bötzinger complex
SL: Superficial layer
USV: Ultrasonic vocalization
